# Regional differences in treatment rates for patients with chronic hepatitis C infection: Systematic review and meta-analysis

**DOI:** 10.1371/journal.pone.0183851

**Published:** 2017-09-06

**Authors:** Philip Vutien, Michelle Jin, Michael H. Le, Pauline Nguyen, Sam Trinh, Jee-Fu Huang, Ming-Lung Yu, Wan-Long Chuang, Mindie H. Nguyen

**Affiliations:** 1 Division of Gastroenterology and Hepatology, Stanford University Medical Center, Palo Alto, California, United States of America; 2 Rush University Medical Center, Chicago, Illinois, United States of America; 3 Hepatobiliary Section, Department of Internal Medicine and Hepatitis Center, Kaohsiung Medical University Hospital, Kaohsiung Medical University, Kaohsiung, Taiwan; Nihon University School of Medicine, JAPAN

## Abstract

**Background & aims:**

Treatment rates with interferon-based therapies for chronic hepatitis C have been low. Our aim was to perform a systematic review of available data to estimate the rates and barriers for antiviral therapy for chronic hepatitis C.

**Methods:**

We conducted a systematic review and meta-analysis searching MEDLINE, SCOPUS through March 2016 and abstracts from recent major liver meetings for primary literature with available hepatitis C treatment rates. Random-effects models were used to estimate effect sizes and meta-regression to test for potential sources of heterogeneity.

**Results:**

We included 39 studies with 476,443 chronic hepatitis C patients. The overall treatment rate was 25.5% (CI: 21.1–30.5%) and by region 34% for Europe, 28.3% for Asia/Pacific, and 18.7% for North America (*p* = 0.008). On multivariable meta-regression, practice setting (tertiary vs. population-based, *p* = 0.04), region (Europe vs. North America *p* = 0.004), and data source (clinical chart review vs. administrative database, *p* = 0.025) remained significant predictors of heterogeneity. The overall treatment eligibility rate was 52.5%, and 60% of these received therapy. Of the patients who refused treatment, 16.2% cited side effects, 13.8% cited cost as reasons for treatment refusal, and 30% lacked access to specialist care.

**Conclusions:**

Only one-quarter of chronic hepatitis C patients received antiviral therapy in the pre-direct acting antiviral era. Treatment rates should improve in the new interferon-free era but, cost, co-morbidities, and lack of specialist care will likely remain and need to be addressed. Linkage to care should even be of higher priority now that well-tolerated cure is available.

## Introduction

Together with chronic hepatitis B, chronic hepatitis C (CHC) is a leading cause of death and disability worldwide.[1] The enormous health cost attributable to viral hepatitis and the availability of effective treatments suggests an important opportunity to improve public health, especially in the case of CHC now that a simple and well-tolerated therapeutic cure is available. As part of a global strategy for eliminating viral hepatitis as a major public health concern by 2030, the World Health Organization (WHO) has set a goal of treating 80% of eligible CHC with antiviral therapy.[2] Unfortunately, treatment rates are far below this number. Several U.S. based studies report treatment rates with pegylated-interferon (PEG-IFN) and ribavirin (RBV) that range from nine to 36%.[3–7] In their report the WHO also estimates that under 1% of treatment eligible CHC patients worldwide have received antiviral therapy.[2]

These low treatment rates are likely due to both PEG-IFN/RBV related toxicities and contraindications as well as systems-level barriers such as medication cost, insurance re-imbursement, and appropriate specialist follow up. Newer direct acting antiviral agents (DAAs) will likely lower barriers related to treatment eligibility and patient/provider willingness to undergo treatment, but systems-level barriers will likely persist.[8, 9] In addition, it is unclear how treatment rates and barriers vary worldwide where patient populations and healthcare practices differ. As CHC becomes a more easily cured disease, it becomes increasingly important to understand where best to direct our resources to improve access to care.

Our aim was to perform a systematic review of available data to estimate treatment rates for CHC worldwide.

## Materials and methods

### Data sources and searches

We performed a systematic review and meta-analysis searching MEDLINE and SCOPUS databases for studies with available treatment rates for CHC patients from January 1991 through March 2016. Articles were queried from MEDLINE using the following search terms: (((hepatitis C[Title] OR HCV[Title]) AND (treatment[Title] OR antiviral[Title])) AND english[Language]) AND (rate[Text Word] OR referral[Text Word] OR duration[Text Word] OR linkage[Text Word] OR specialist[Text Word] OR intake[Text Word] OR multivariate[Text Word]).

Articles were queried from SCOPUS with the following search terms: (‘hepatitis C’ OR ‘HCV’) AND (‘treatment’). Non-English articles were excluded in both queries.

We also conducted a manual search of abstracts using the term ‘hepatitis C’ from annual international scientific meetings held in the 2 years preceding the literature search date and by the American Association for the Study of Liver Diseases (AASLD), Digestive Disease Week, the Asian Pacific Study of the Liver, and the European Association for the Study of the Liver (EASL). All data were collected according to the Preferred Reporting Items for Systematic Reviews and Meta-Analyses (PRISMA) guidelines.[10]

### Inclusion and exclusion criteria

We included original studies with ≥ 25 CHC patients with available antiviral treatment rates. Treatment was defined by receipt of interferon, PEG-IFN, RBV, or DAA-based therapies. Exclusion criteria included studies of populations from randomized control trials and studies of specialized populations including renal hemodialysis centers, human immunodeficiency virus (HIV) clinics, or drug rehabilitation programs. We also excluded studies of cohorts with high rates (≥ 10%) of HIV and/or hepatitis B co-infection. In the case of studies with overlapping patient populations, we excluded abstracts at major liver meetings if there was a corresponding published manuscript. If multiple manuscripts were published from a similar patient database then we included the study with the largest number of patients.

### Study selection and study extraction

**Fig 1:**
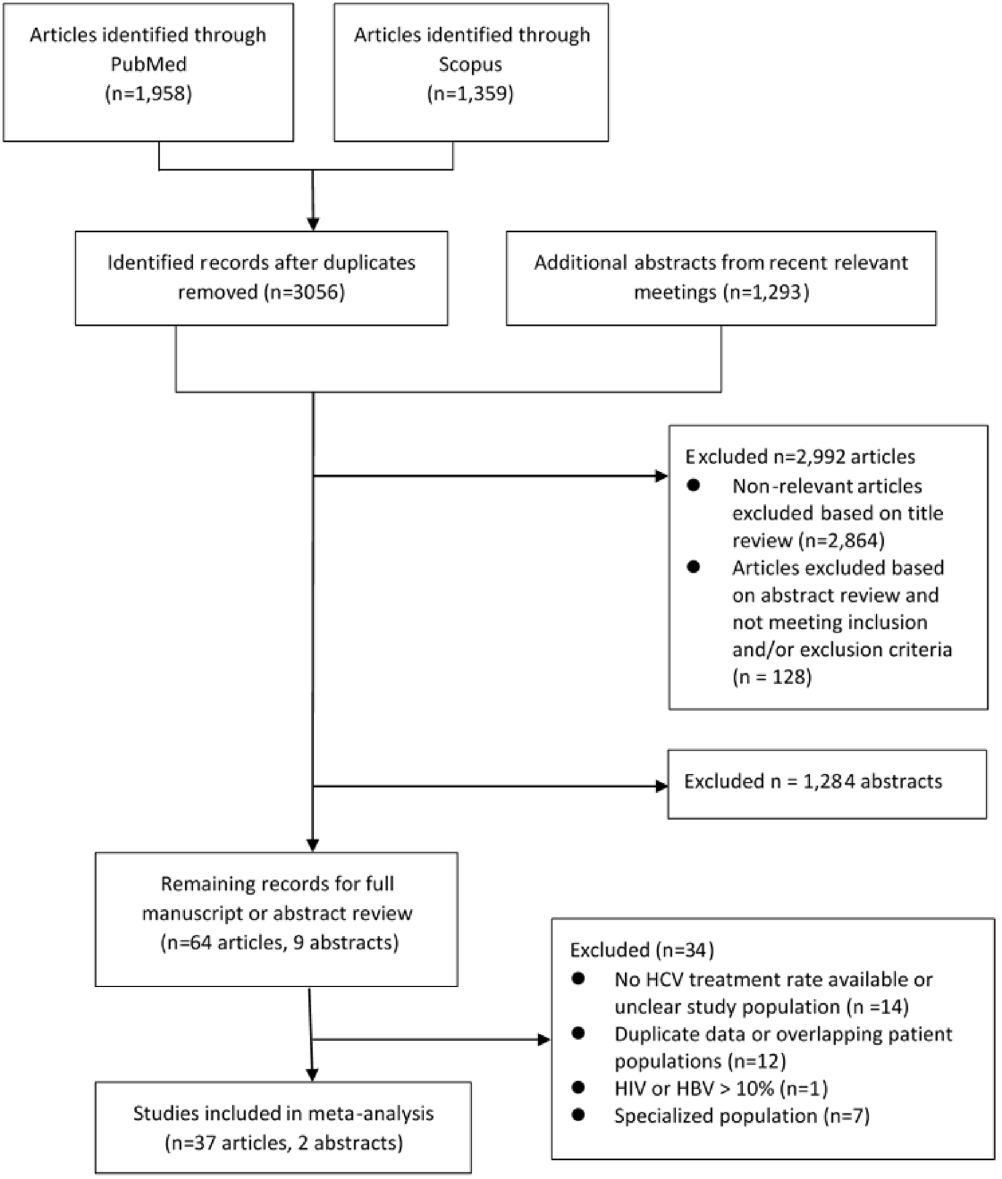
PRISMA flow diagram.

Four authors (PV, MJ, PN, ML) independently assessed study titles and abstracts for eligibility (Fig 1). Studies that were considered eligible were then selected for full-text review. The authors then extracted individual study characteristics, patient treatment and eligibility rates, and patient medical and demographic data using a standardized case report form. Any discrepancies were resolved by discussion with the authors including the senior author (MN).

### Study definitions

Population-based studies were defined as those that queried patients from national or region-wide databases/registries and did not recruit from a distinct number of clinics or hospitals. Advanced fibrosis was defined by the presence of cirrhosis or by a score of F3 or F4 on the Metavir scale.[11] Studies were further characterized by type of CHC treatment data collection: patient questionnaires, individual clinical chart review, and electronic query of administrative databases (i.e. pharmaceutical prescription or national insurance databases).

### Study quality assessment

Study quality was assessed using a scoring system adapted after a modified Newcastle-Ottawa Quality Assessment scale.[12] Two authors (MLY, JFH) scored each study by three criteria: selection (maximum of five points assessing representativeness of the study population, sample size, and ascertainment of HCV exposure), comparability (maximum of one point), and outcome (maximum of three points assessing for reliability of HCV treatment and the statistical test used). As defined by prior studies, a score of seven or more was considered a “good” quality study.[13]

### Statistical analysis

We analyzed pooled treatment rates with corresponding 95% confidence intervals (CIs) using random-effects models and odds ratios (OR) for sub-analyses comparing groups within studies. We assessed for study heterogeneity with χ^2^-based Cochrane Q-statistic with *p* ≤ 0.1 and I^2^ ≥ 50% as measures for substantial study heterogeneity in our models. Multiple separate meta-analyses were performed on study-level characteristics including study region, quality assessment scores, type of therapy studied, patient recruitment period, and data collection methodology. Multivariable random-effects meta-regression on study-level characteristics were also performed to explain any observed heterogeneity in CHC treatment rates. All statistical tests were were performed using Comprehensive Meta-Analysis, version 3 (Biostat, Englewood, New Jersey, USA).

## Results

Our literature search identified 1,958 articles from MEDLINE, 1,359 articles from SCOPUS and 1,293 abstracts (Fig 1). After reviewing titles and abstracts, the full texts of 73 studies (64 manuscripts and nine abstracts) were closely evaluated for eligibility.

As shown in Table 1, a total of 39 studies with 476,443 CHC patients (37 articles and 2 abstracts) met eligibility criteria and were included in our primary meta-analyses.[3, 4, 6, 7, 14–48] Most studies were from North America (19/39, 49%) or Europe (11/39, 28%). Eight (21%) were from the Asia or Pacific regions. By setting, approximately half of the studies were from tertiary/referral centers (19/40, 47.5%) and close to half were from population-based settings (17/40, 42.5%). Most studies (28/39, 71.7%) collected treatment information through clinical chart review. Seven studies (18%) collected treatment prescription via electronic data extraction of large administrative databases and 4 studies (10.3%) through patient questionnaires.

**Table 1:** Characteristics of included studies.

First Author, Year	Country	Study setting	Inclusion years	Number of patients	Therapy examined	HCV treatment data collection
Grebely, 2011	Australia	Population based*	2008	634	PEG-IFN^†^ + RBV^‡^	Patient questionnaire
Stoove, 2005	Australia	Population based	2000–2002	659	IFN§ or PEG-IFN + RBV	Patient questionnaire
Delwaide, 2005	Belgium	Tertiary referral	1996–2003	299	IFN or PEG-IFN + RBV	Chart review
Vigani, 2008	Brazil	Tertiary referral	2003–2006	275	PEG-IFN + RBV	Chart review
Moirand, 2007	Canada	Tertiary referral	2001–2002	635	IFN or PEG-IFN + RBV	Chart review
Yau, 2015	Canada	Tertiary referral	2008–2013	164	PEG-IFN + RBV +/- BOC^¶^ or TVL^║^	Chart review
Yan, 2010	China	Tertiary referral	2000–2009	303	IFN or PEG-IFN + RBV	Chart review
Feillant, 2016	France	Tertiary referral	2013	255	PEG-IFN + RBV	Chart review
Kutala, 2015	France	Tertiary referral	2000–2010	685	IFN or PEG-IFN + RBV	Chart review
Kittner, 2014	Germany	Tertiary referral	2011–2012	307	PEG-IFN + RBV + BOC or TVL	Chart review
Gupta, 2015	India	Tertiary referral	2008–2014	530	PEG-IFN + RBV	Chart review
Stroffolini, 2010	Italy	Tertiary referral	2009	534	PEG-IFN + RBV	Chart review
Vukotic, 2015	Italy	Population based	2009–2010	1118	PEG-IFN + RBV	Chart review
Mizui, 2007	Japan	Population based	1991–2001	1019	IFN or PEG-IFN + RBV	Chart review
Lee, 2016	Korea	Tertiary referral	2007–2012	759	PEG-IFN + RBV	Chart review
Toresen, 2014	Norway	Tertiary referral	2007–2010	233	PEG-IFN + RBV	Chart review
Crespo, 2015	Spain	Mixed primary care, tertiary referral	2012	769	PEG-IFN + RBV	Chart review
Hsu, 2015	Taiwan	Population based	1997–2011	194506	IFN or PEG-IFN + RBV	Electronic query
Yu (community), 2015	Taiwan	Community based	2012–2013	586	PEG-IFN + RBV	Patient questionnaire
Yu (specialist), 2015	Taiwan	Tertiary referral	2012–2013	3045	PEG-IFN + RBV	Patient questionnaire
Howes, 2016	United Kingdoms	Population based	2010–2013	197	PEG-IFN + RBV	Chart review
Mcdonald, 2014	United Kingdoms	Population based	1996–2009	5736	IFN or PEG-IFN + RBV	Chart review
Tait, 2010	United Kingdoms	Population based	1994–2008	1012	IFN or PEG-IFN + RBV	Chart review
Chen, 2013	United States	Tertiary referral	2011–2012	487	PEG-IFN + RBV + BOC or TVL	Chart review
Chirikov, 2015	United States	Population based	2006–2008	1936	IFN or PEG-IFN + RBV	Electronic query
Clark, 2012	United States	Tertiary referral	Not available	212	PEG-IFN + RBV	Chart review
Cozen, 2013	United States	Tertiary referral	1992–2007	358	IFN or PEG-IFN + RBV	Chart review
Gundlappali, 2015	United States	Population based	2004–2009	101,444	PEG-IFN + RBV	Electronic query
Livingston, 2012	United States	Tertiary referral	2003–2007	240	PEG-IFN + RBV	Chart review
Markowitz, 2005	United States	Population based	1996–2015	5135	IFN or PEG-IFN + RBV	Electronic query
Moorman, 2013	United States	Population based	2006–2008	8810	PEG-IFN + RBV	Chart review
Morrill, 2005	United States	Community based	2001–2004	208	IFN or PEG-IFN + RBV	Chart review
Narasimhan, 2006	United States	Tertiary referral	1998–2002	433	IFN or PEG-IFN + RBV	Chart review
Nguyen, 2014 (Abstract)	United States	Tertiary referral	1999–2014	9330	Dual, triple, and DAA** based therapies	Chart review
Nyberg, 2014 (Abstract)	United States	Population based	2002–2012	51984	PEG-IFN + RBV	Electronic query
Schaeffer, 2015	United States	Tertiary referral	2006–2011	129	PEG-IFN + RBV	Chart review
Shatin, 2004	United States	Population based	1997–1999	3259	IFN + RBV	Electronic query
Vutien, 2016	United States	Population based	2009–2013	76849	PEG-IFN + RBV +/- BOC or TVL	Electronic query
Yawn, 2008	United States	Population based	1990–2005	626	IFN or PEG-IFN + RBV	Chart review
Younossi, 2013	United States	Population based	2001–2010	203	IFN or PEG-IFN + RBV	Pt questionnaire

*Population-based studies were those that queried patients from national or region-wide databases/registries

^†^PEG-IFN—Pegylated-interferon

^‡^RBV—Ribavirin

^§^IFN—Interferon

^¶^BOC—Boceprevir

^║^TVL—Telaprevir

**DAA therapies include sofosbuvir, simeprevir, and ledipasvir

Based on the modified Newcastle-Ottawa quality score for cross-sectional studies, the mean score of our 39 studies was seven (S1 Table). Over half of the studies (22/39, 56%) were considered good quality, as defined by a quality score of seven or higher.[13]

### Pooled CHC treatment rates and by patient-level characteristics across studies

**Fig 2:**
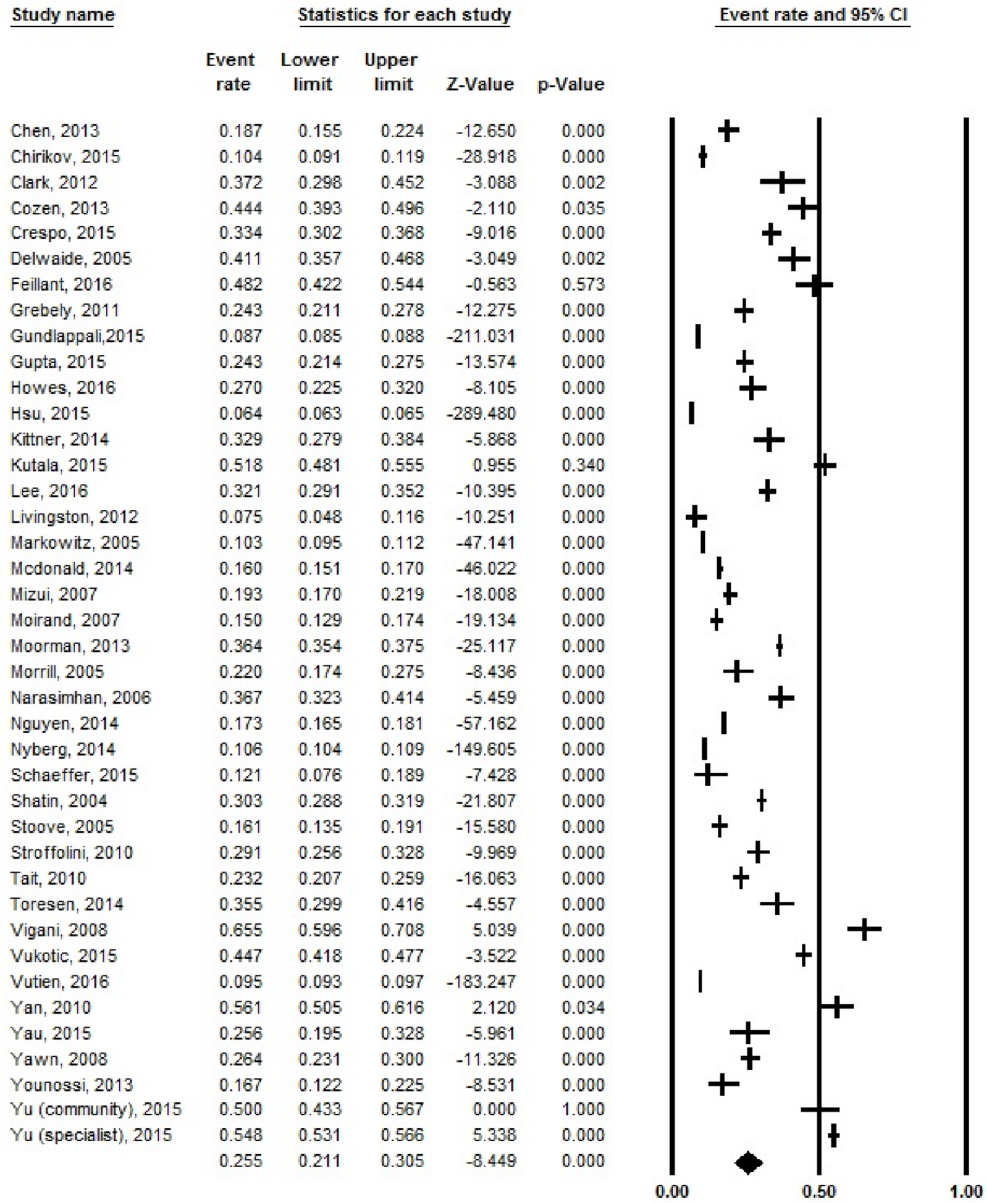
Overall pooled treatment rate for all patients with chronic hepatitis C.

The overall pooled treatment rate was 25.5% (CI: 21.1–30.5%) and there was significant heterogeneity (I^2^ = 99.8, *p* < 0.001) (Fig 2). On a sub-analysis of eight studies with available data, HCV genotype 1 were less likely to be treated than non-HCV genotype 1 patients (OR = 0.7, CI: 0.63–0.78, *p* < 0.001) (S2 Table). There was no significant difference in treatment rates for patients with advanced fibrosis vs. without fibrosis (OR = 1.27, *p* = 0.39) or males vs. females (OR = 0.88, *p* = 0.14).

### Treatment rates by region

**Fig 3:**
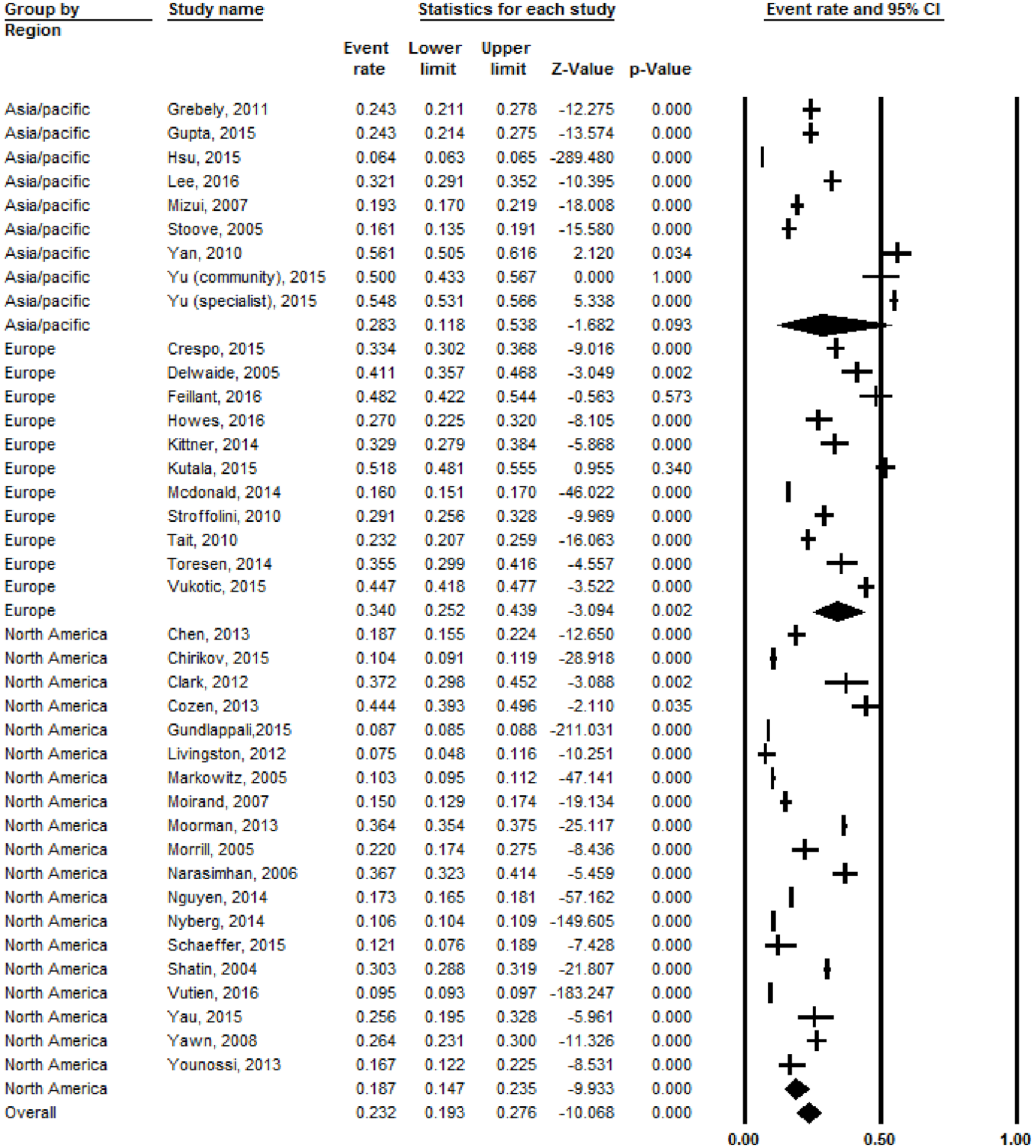
Pooled treatment rates for patients with chronic hepatitis C, by region.

By region, studies from Europe had the highest treatment rate (34%, 95% CI: 25.2–43.9%) compared to the Asia/Pacific region (28.3%, 95% CI: 11.8–53.8%) and North America (18.7%, 95% CI: 14.7–23.5%; *p* = 0.008) (Fig 3). In this meta-analysis Vigani et al. was excluded as it was the only study from South America.[43] When comparing separate regions, only the difference between Europe versus North America was statistically significant (*p* = 0.002). There were similar pooled treatment rates for studies from single-payer reimbursement systems (24%) as compared to multi-payer ones (19%, *p* = 0.53, data described in text only).

Vigani et al. was excluded as it was the only study from South America.

### Treatment rates by practice setting

Treatment rates were significantly higher in the 19 tertiary referral-based studies compared to those from 17 population-based studies (31.7% of referral vs. 17.7% of population-based studies, *p* = 0.003).

### Treatment rates by data collection methods

Treatment rates were highest when studies collected treatment data by medical chart review (29.8%) and patient questionnaire (30.3%) as done in many studies from referral centers, as compared to electronic query and extraction of population-based administrative databases (11.1%, *p* < 0.001).

### Treatment rates by therapy type

Studies that examined only CHC patients treated with triple therapies including boceprevir or telaprevir did not report higher treatment rates than those that reported treatment rates with dual therapies (32% for triple therapy vs. 25.2% for dual therapy studies, *p* = 0.61).

### Random-effects meta-regression of study characteristics

Table 2 shows the meta-regression of five study-level factors (practice setting, region, quality assessment score, therapy type, and type of data collection for CHC treatment) testing for sources of heterogeneity of HCV treatment rates.

**Table 2:** Meta-regression for predictors for antiviral therapy for chronic hepatitis C.

	Univariate analysis		Multivariate analysis	
*Predictors for treatment*	*Unadjusted Coefficient (95% CI)*	*P value*	*Adjusted Coefficient (95% CI)*	*P value*
Tertiary center vs. population based	0.77 (0.33–1.21)	< 0.001	0.41 (0.02–0.8)	0.04
Region				
North America	Referent	-	-	-
Asia/Pacific	0.53 (-0;12–1.2)	0.11	0.28 (-0.17–0.72)	0.22
Europe	0.8 (0.18–1.43)	0.011	0.61 (0.2–1)	0.004
Quality assessment score of ≥ 7 vs. < 7	-0.22 (-0.7–0.24)	0.34	-	-
Triple vs. dual interferon-based therapy	0.33 (-0.62–0.1.3)	0.69	-	-
Ascertainment of chronic hepatitis C diagnosis				
Chart review	Referent	-	Referent	-
Electronic query	0.03 (-0.4–0.46)	<0.001	-0.6 (-1.1–0.07)	0.025
Patient questionnaire	0.03 (-0.4–0.46)	0.9	0.18 (-0.4–0.78)	0.57

On meta-regression model practice setting (tertiary vs. population-based, *p* = 0.04), region (Europe vs. North America *p* = 0.004), and treatment data collection type (chart review vs. electronic query, *p* = 0.025) remained significant predictors of heterogeneity.

### Treatment eligibility rates

**Fig 4:**
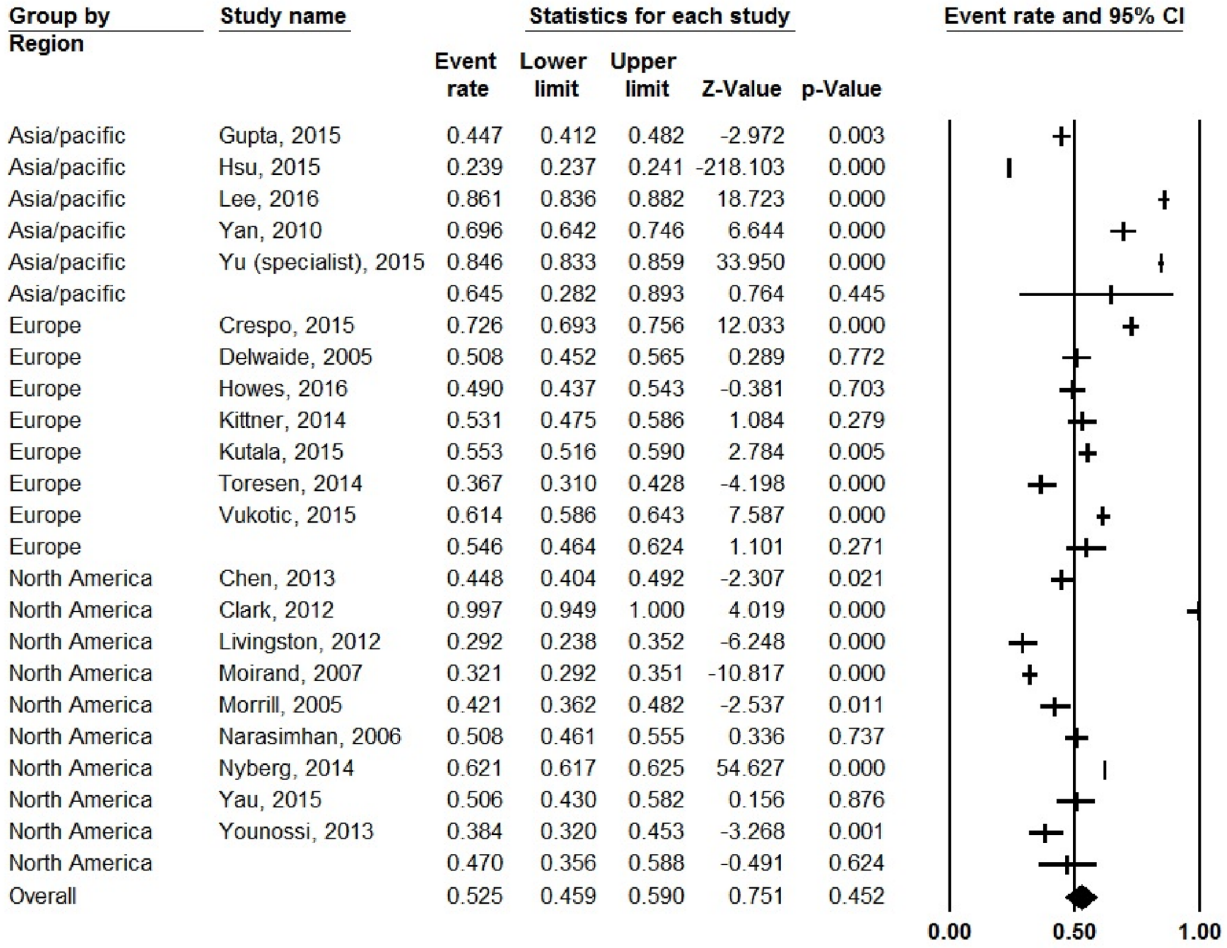
Pooled treatment eligibility rates for patients with chronic hepatitis C, by region.

Analysis of twenty-one studies with available data showed a pooled eligibility rate of 52.5% (CI: 45.9–59%), and 60% (CI: 49.2–69.9%) of eligible patients were treated. There was no statistically significant difference in eligibility rates by region (64.5% for Asia/Pacific region, 54.6% for Europe, and 47% for North America, *p* = 0.48) (Fig 4). On sub-analysis of treatment rates among eligible patients by region, studies from Europe had higher treatment rates (76.8%), while studies from the Asia/Pacific (53.2%), and North America had lower rates (42.2%, *p* for overall model = 0.01) (S1 Fig).

### Reasons for treatment ineligibility and treatment refusal

Loss to follow up or lack of referral to HCV specialists was the most common reason for no treatment (14.6%, CI: 5.5–33.6%) (Table 3). Other reasons include normal liver tests or lack of significant fibrosis (8.6%, CI: 4.1–17.2%), medical contraindications (11.4%, CI: 6–20.7%), psychiatric contraindications (3.6%, CI: 2.5–5.3%), and active substance abuse (2.8%, CI: 1.2–6.4%). The most common reasons for treatment refusal by eligible patients was concern for treatment side effects (16.2%, CI: 13.3–19.5%), cost or insurance issues (13.8%, CI: 6.4–27.2%), and waiting for better treatment (12.2%, CI: 8.2–17.6%) (Table 4).

**Table 3:** Pooled treatment ineligibility rates for patients with chronic hepatitis C, by reasons for ineligibility.

*Treatment ineligibility criteria*	*Treatment ineligibility rate (95% CI)*	*No*. *of studies included*
Normal liver tests or lack of fibrosis	8.6% (4.1–17.2%)	9
Medical comorbidities (includes decompensated liver disease)	11.4% (6–20.7%)	16
Psychiatric comorbidities	3.6% (2.5–5.3%)	13
Substance abuse	2.8% (1.2–6.4%)	9
Loss to follow up	14.6% (5.5–33.6%)	13

**Table 4:** Pooled patient refusal rates in treatment-eligible patients with chronic hepatitis C, by reasons for refusal.

*Reason for patient refusal*	*Patient refusal rates(95% CI)*	*No*. *of studies included*
Side effects	16.2% (13.3–19.5%)	5
Cost or insurance issues	13.8% (6.4–27.2%)	6
Waiting for better treatment	12.2% (8.2–17.6%)	5

## Discussion

In this systematic review, we found that only one-quarter (25.5%) of CHC patients received antiviral therapy. Fifty-two percent of patients were eligible for treatment and only 60% of these eligible patients received antiviral therapy. In addition, for the 47% of patients who were not eligible for treatment, we found the most common reasons for treatment ineligibility were loss to follow up or lack of referral to HCV specialists. This overall low treatment rate is well below the WHO’s goal treatment rate of 80% and demonstrates the importance of proper referral and follow up for CHC patients to receive treatment. [4, 6, 25, 49, 50] While many of the reasons for treatment ineligibility were specific to PEG-IFN + RBV, this issue in linkage-to-care is multifactorial and related in part to the asymptomatic nature of CHC but also to a lack of the ability to provide extensive pre-treatment workup including serologic testing and assessment for liver fibrosis and inflammation.[6, 45, 49–51]

We also found no statistical difference in treatment rates with PEG-IFN + RBV combined with first generation DAAs boceprevir or telaprevir (32%) compared to those examining PEG-IFN + RBV alone (25%, *p* = 0.61). Despite the improved sustained viral response rates, the similar treatment rates with triple therapy were likely reflective of the significant barriers inherent to PEG-IFN + RBV. The use of the well tolerated newer all-oral DAA therapies will likely diminish the barriers related to treatment eligibility and provider/patient acceptance, especially those related to medical and/or psychiatric contraindications.

Another significant barrier to treatment for eligible patients in this study was cost and insurance related. An estimated 14% of treatment-eligible patients declined therapy because of cost or were denied insurance coverage. This is an important point as the evolution of the newer and more expensive interferon free DAA’s continues and more drugs enter the market. With the current costs of all-oral DAAs well exceeding that of PEG-IFN-based therapies, the non-treatment rate due to insurance approval and cost may actually rise compared to that from the PEG-IFN era even in high income countries.[52, 53]

Furthermore, our pooled treatment rates, while low, are still likely an underestimation of the overall CHC treatment rate considering that many CHC patients remain undiagnosed. As we now have highly effective DAAs, it becomes even more important to identify patients with CHC early in their disease course and also issues in our linkage to specialist care or primary providers comfortable in the management of these patients.

On our meta-analysis of treatment rates by region, Europe had the highest treatment rate (34%), followed by the Asia/Pacific region (28.3%), and finally North America (18.7%). Some of these differences may be attributable to study methodologies which were also important predictors of treatment rate heterogeneity. North America had the highest proportions of population-based studies and also studies that queried HCV treatment electronically both found to be predictors of treatment rate heterogeneity. Population-based studies may have lower treatment rates due to the inclusion of all-comers with CHC: patients evaluated in community clinics and emergency rooms who are not referred to specialists.

However, on our final meta-regression model adjusting for these study methodologies, we found that region (Europe vs. North America) remained a significant source of heterogeneity (*p* = 0.004). In addition, on a separate analysis, treatment eligibility rates were not significantly different among the 3 geographic regions (47% for North America vs. 65% for Asia/Pacific and 55% for Europe, overall *p* = 0.48). This suggests that some of the barriers are specific to North America and include insurance reimbursement criteria, and patient-physician preferences.

Small, but potentially significant differences between international guidelines for the use of PEG-IFN and RBV based therapies may also have affected treatment rates. While all three major international guidelines (EASL, AASLD, and APASL) recommend against treating decompensated cirrhotics with interferon-based therapies, the guidelines from AASLD further specify acceptable laboratory parameters: total serum bilirubin < 1.5 g/dL, International Normalized Ratio < 1.5, platelet count < 75,000, and serum albumin > 3.4).[54] In the guidelines published by EASL and APASL many of these laboratory criteria are absent or considered relative contraindications to treatment.[55, 56] The AASLD guidelines also strongly recommend a baseline liver biopsy to assess baseline liver inflammation and fibrosis prior to initiating treatment.[54] In contrast the guidelines from APASL and EASL, published in subsequent years, included other non-invasive methods including transient elastography and blood marker panels as potential substitutes for liver biopsy.[55, 56]

Based on this systematic review, there are no studies that directly examined treatment rates and barriers to care with the newer DAA therapies and this is an area that will require further research. One paper, presented by Moon et al., reported a significant increase in treatment prescriptions in 2015 compared to the prior PEG-IFN years. The investigators attributed this large increase to the introduction of 2nd generation DAA therapies into the CHC treatment armament.[57] This is likely a result of the lower treatment threshold of DAA-based therapies: the revised recommendations for DAA therapy from EASL, AASLD, and APASL have recommended considering treatment for all CHC patients including those with decompensated liver disease.[58–60]

Our study does have a few limitations. Several of the sub-analyses included fewer studies so the results should be interpreted with caution. There was also high heterogeneity among our studies, which is due to the variety of patient populations, regional practices, time period of the study, and sample sizes. To address this, we analyzed by subgroups and also attempted to control for confounders through the use of a multivariable meta-regression model. Finally, our results may not be generalizable to certain regions such as Africa and the Middle East due to the lack or relative underrepresentation of studies from these regions. This is concerning because these regions have the largest HCV disease burden in the world.[61] A recent meta-analysis examining operational interventions to enhance chronic viral hepatitis testing and linkage to care found that several simple, inexpensive operational interventions can improve engagement and retention in the cascade of care of patients with chronic viral hepatitis, but further operational research is needed in these regions.[62]

Our study is the first systematic review to examine HCV treatment rates for all geographic regions with available data. We found that treatment rates were suboptimal with only 25.5% overall, and only 60% of CHC patients worldwide, who met treatment criteria and did not have any medical or psychiatric contraindication, received treatment before the availability of IFN-free regimens. While these low treatment rates are partly attributable to PEG-IFN and ribavirin, further research efforts are needed to identify and quantify other treatment barriers that may persist in this IFN-free DAA era and especially those related to cost, insurance authorization, and lack of linkage to care with providers familiar with the management of patients with CHC.

## Supporting information

S1 Table Newcastle-Ottawa quality assessment scores for individual studies. (DOCX)

S2 Table Predictors for treatment by patient-level factors across studies. (DOCX)

S1 Fig Pooled treatment rates for treatment eligible patients with chronic hepatitis C, by region. (PDF)

S1 Checklist PRISMA checklist. (DOCX)

S1 Database (XLSX)

## Abbreviations

AASLD: American Association for the Study of Liver Diseases
CI: Confidence interval
CHC: Chronic hepatitis C
DAA: Direct acting antiviral
EASL: the European Association of the Study of the Liver
HBV: Hepatitis B
HIV: Human immunodeficiency virus
OR: Odds ratios
PEG-IFN: Pegylated-interferon
PRISMA: Preferred Reporting Items for Systematic Reviews and Meta-Analyses
RBV: Ribavirin
WHO: World Health Organization
