# Supplementary material for: Regional differences in treatment rates for patients with chronic hepatitis C infection: Systematic review and meta-analysis
Source: PLoS One. 2017 Sep 6;12(9):e0183851. doi: 10.1371/journal.pone.0183851 (PMC5587234; doi:10.1371/journal.pone.0183851)
Supplement: S1 Table — (DOCX) [file pone.0183851.s001.docx]

S1 Table. Newcastle-Ottawa quality assessment scores for individual studies

| **Author, Year** | **Selection score**  **(5 points)**^*^ | **Comparability score**  **(1 point)**^†^ | **Outcome score**  **(3 points)**^‡^ | **Total**  **(9 points)** |
| --- | --- | --- | --- | --- |
| Chen, 2013 | 4 | 1 | 3 | 8 |
| Chirikov, 2015 | 4 | 0 | 2 | 6 |
| Clark, 2012 | 3 | 1 | 3 | 7 |
| Cozen, 2013 | 2.5 | 1 | 3 | 6.5 |
| Crespo, 2015 | 5 | 1 | 3 | 9 |
| Delwaide, 2005 | 2.5 | 1 | 3 | 6.5 |
| Feillant, 2016 | 4 | 1 | 3 | 8 |
| Grebely, 2011 | 2.5 | 1 | 1.5 | 5 |
| Gundlappali,2015 | 4 | 1 | 3 | 8 |
| Gupta, 2015 | 3.5 | 1 | 3 | 7.5 |
| Howes, 2016 | 5 | 0 | 1.5 | 6.5 |
| Hsu, 2015 | 4 | 1 | 2 | 7 |
| Kittner, 2014 | 3 | 1 | 3 | 7 |
| Kutala, 2015 | 2.5 | 1 | 3 | 6.5 |
| Lee, 2016 | 5 | 1 | 3 | 9 |
| Livingston, 2012 | 3 | 0 | 2.5 | 5.5 |
| Markowitz, 2005 | 5 | 1 | 3 | 9 |
| Mcdonald, 2014 | 4 | 1 | 3 | 8 |
| Mizui, 2007 | 5 | 1 | 3 | 9 |
| Moirand, 2007 | 2.5 | 0 | 3 | 5.5 |
| Moorman, 2013 | 5 | 0 | 3 | 8 |
| Morrill, 2005 | 2.5 | 0 | 2.5 | 5 |
| Narasimhan, 2006 | 2.5 | 1 | 3 | 6.5 |
| Nguyen, 2014 | 4 | 0 | 3 | 7 |
| Nyberg, 2014 | 5 | 1 | 2 | 8 |
| Schaeffer, 2015 | 3 | 1 | 3 | 7 |
| Shatin, 2004 | 4 | 0 | 2 | 6 |
| Stoove, 2005 | 4 | 0 | 2 | 6 |
| Stroffolini, 2010 | 4 | 1 | 3 | 8 |
| Tait, 2010 | 5 | 0 | 2.5 | 7.5 |
| Toresen, 2014 | 3 | 1 | 3 | 7 |
| Vigani, 2008 | 3 | 0 | 2 | 5 |
| Vukotic, 2015 | 4 | 1 | 3 | 8 |
| Vutien, 2016 | 4 | 1 | 3 | 8 |
| Yan, 2010 | 2.5 | 1 | 3 | 6.5 |
| Yau, 2015 | 2 | 1 | 3 | 6 |
| Yawn, 2008 | 3 | 0 | 2.5 | 5.5 |
| Younossi, 2013 | 3 | 0 | 1.5 | 4.5 |
| Yu (community), 2015 | 5 | 1 | 2 | 8 |
| Yu (specialist), 2015 | 5 | 1 | 2 | 8 |

^*^Selection score was based on representativeness of the study population (2 points), sample size (1 point) and ascertainment of HCV exposure (2 points)

^†^Comparability score was based on whether or not the study stratified treatment rates by cirrhosis, HCV genotype, or age (1 point)

^‡^Outcome score was based on assessment of HCV treatment (2 points) and statistical test used (1 point)
