# Supplementary material for: Regional differences in treatment rates for patients with chronic hepatitis C infection: Systematic review and meta-analysis
Source: PLoS One. 2017 Sep 6;12(9):e0183851. doi: 10.1371/journal.pone.0183851 (PMC5587234; doi:10.1371/journal.pone.0183851)
Supplement: S2 Table — (DOCX) [file pone.0183851.s002.docx]

Supplemental table 2. Predictors for treatment by patient-level factors across studies

| ***Factor*** | ***Odds ratio for treatment*** | ***Number of studies*** | ***P value*** |
| --- | --- | --- | --- |
| Cirrhosis vs. no cirrhosis | 1.27 (0.74 – 2.2) | 9 | 0.39 |
| Male vs. female gender | 0.88 (0.74 – 1.04) | 17 | 0.14 |
| HCV genotype 1 vs. non-1 genotype | 0.7 (0.63 – 0.78) | 8 | < 0.001 |
