# Supplementary material for: Regional differences in treatment rates for patients with chronic hepatitis C infection: Systematic review and meta-analysis
Source: PLoS One. 2017 Sep 6;12(9):e0183851. doi: 10.1371/journal.pone.0183851 (PMC5587234; doi:10.1371/journal.pone.0183851)

S1 Figure. Pooled treatment rates for treatment eligible patients with chronic hepatitis C, by region

Group by Region	Study name	Event rate	Lower limit	Upper limit	Z-Value	p-Value
		Statistics for each study				
Asia/pacific	Gupta, 2015	0.545	0.492	0.596	1.662	0.097
Asia/pacific	Hsu, 2015	0.267	0.263	0.271	-96.324	0.000
Asia/pacific	Lee, 2016	0.373	0.339	0.408	-6.927	0.000
Asia/pacific	Yan, 2010	0.806	0.747	0.854	8.174	0.000
Asia/pacific	Yu (specialist), 2015	0.648	0.629	0.666	14.799	0.000
Asia/pacific		0.532	0.315	0.738	0.280	0.780
Europe	Crespo, 2015	0.461	0.420	0.502	-1.861	0.063
Europe	Delwaide, 2005	0.809	0.739	0.864	6.999	0.000
Europe	Howes, 2016	0.552	0.475	0.626	1.321	0.186
Europe	Kittner, 2014	0.620	0.543	0.691	3.025	0.002
Europe	Kutala, 2015	0.937	0.907	0.957	12.773	0.000
Europe	Toresen, 2014	0.968	0.906	0.990	5.815	0.000
Europe	Vukotic, 2015	0.728	0.693	0.760	11.474	0.000
Europe		0.768	0.625	0.868	3.418	0.001
North America	Chen, 2013	0.417	0.354	0.484	-2.427	0.015
North America	Clark, 2012	0.372	0.298	0.452	-3.088	0.002
North America	Livingston, 2012	0.257	0.168	0.372	-3.879	0.000
North America	Moirand, 2007	0.467	0.412	0.523	-1.143	0.253
North America	Morrill, 2005	0.523	0.429	0.615	0.479	0.632
North America	Narasimhan, 2006	0.723	0.660	0.778	6.361	0.000
North America	Nyberg, 2014	0.171	0.167	0.176	-106.698	0.000
North America	Yau, 2015	0.506	0.400	0.612	0.110	0.913
North America	Younossi, 2013	0.436	0.331	0.547	-1.129	0.259
North America		0.422	0.260	0.603	-0.837	0.403
Overall		0.600	0.492	0.699	1.816	0.069

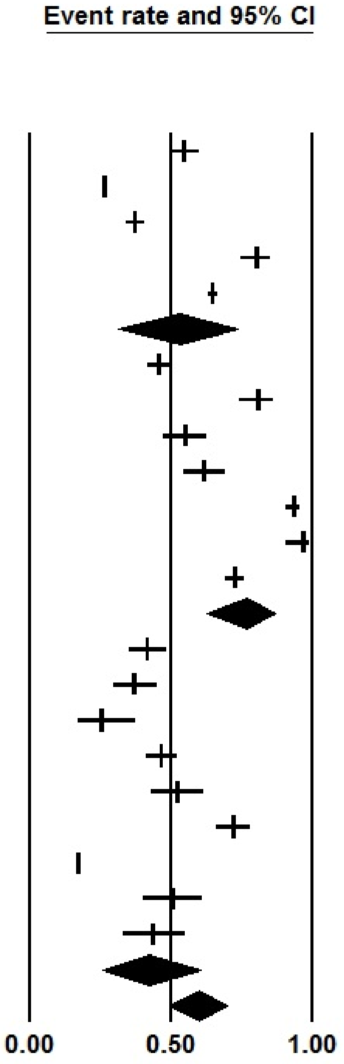

Supplement: S1 Fig — (PDF) [file pone.0183851.s003.pdf]
